# Factors Associated with Length of Stay in Psychiatric Inpatient Services in London, UK

**DOI:** 10.1007/s11126-017-9498-7

**Published:** 2017-04-03

**Authors:** Loveday Newman, Victoria Harris, Lauren J Evans, Alison Beck

**Affiliations:** 1 Psychology and Psychotherapy Department, South London and Maudsley NHS Foundation Trust, London, UK; 2 Biostatistics Department, Institute of Psychiatry, Psychology and Neuroscience at King’s College London, London, UK; 3 M/4/1/0240, First Floor Administration Building, Maudsley Hospital, Denmark Hill, London, SE5 8AZ UK

**Keywords:** Inpatient services, Length of stay, Predictors, Hospitalisation

## Abstract

The purpose of this research was to identify factors associated with lengthy stays in psychiatric hospital in a UK mental health trust. A multiple regression using a multiple imputation method to deal with missing data was performed on inpatient admissions data over a four-year period for 7653 individuals. Factors associated with a longer length of stay included gender (being male), ethnicity (being Asian/Asian British, Black/Black British, or having a mixed background compared to being White/White British), accommodation status (being homeless, or in accommodation with support), primary diagnostic group of psychosis (F20–29), and number of care coordinators. Marital status was not found to be associated with length of stay in this sample. Length of stay is likely to be multifactorially determined, and more similar studies examining factors associated with length of hospital stay are needed to understand the operation of psychiatric services.

## Introduction

Despite a decline of length of stay (LOS) in psychiatric hospitals [1], some patients continue to experience lengthy stays in psychiatric inpatient units. Current UK mental health policy advocates short hospital stays and a focus on care in the community [2]. Current evidence suggests that short hospital stays do not lead to a ‘revolving door’ pattern of admissions [3–5], although others have found a link between short hospital stays and an increased likelihood of readmission [6].

Leff & Trieman [7] investigated social and clinical outcomes of long-stay patients who were discharged to community care in the 1990s following the closure of two psychiatric hospitals in London. Symptoms and social behaviour problems largely remained stable at follow-up 1 and 5 years later. In addition, individuals were shown to gain friends, confidants as well as domestic and community skills. When asked, 84% of respondents said they wanted to remain in their current homes. Although this study had a high rate of attrition (only 53% of the sample were followed up at 5 years), these results suggest that the quality of life of long-stay patients is not seriously diminished by being discharged from hospital, and may even be beneficial.

Different demographic and clinical characteristics have been previously been found to be associated with lengthy stays in hospital. These include a diagnosis of psychosis [8, 9], having an involuntary admission [10], severity of illness [11], receiving ECT [10], being unemployed [12, 13], unstable accommodation status [11, 14], homelessness [15] and female gender [8, 9].

Although many studies have examined factors relating to LOS, there are few up-to-date studies conducted in the UK. With the exception of Tulloch et al. [15], large-scale UK studies comprising large samples and extended study periods use data that is now over 20 years’ old [8, 16]. In their review of US studies on factors predicting LOS, Tulloch et al. [9] found that the influence of some factors was only apparent with very large samples. This might be due to the frequent lack of variation seen amongst inpatient psychiatric populations on factors such as employment and marital status [17].

As suggested by Tulloch et al. [9], more up-to-date research on LOS is needed to understand determinants of LOS in countries outside the US, where most research on this topic has been conducted. The aim of this study is therefore to examine factors that predict LOS in order to better understand psychiatric care use in a large UK mental health trust.

## Methods

### Description of the UK National Health Service

The National Health Service (NHS) is a publicly funded health service and provides free, universal healthcare to the UK general population. The English NHS is divided into trusts, which are divisions within the service generally serving a particular geographical location. Mental health trusts are part of secondary care and since 2013, are funded by Clinical Commissioning Groups (CCG), which are clinically led statutory bodies that plan and commission healthcare services at the local level. They can plan and commission any service provider that meets NHS standards and costs.

Current policy recommends increased community-based care, as opposed to lengthy stays in hospital [2]. The Mental Health Act 1983 and the more recent Mental Health Act 2007 gives approved mental health professionals the power to admit and detain an individual to hospital against their will, if it is considered the best and most appropriate way to ensure the individual receives the right care and treatment.

### Description of Current Setting

We examined admissions to adult psychiatric inpatient services to a large UK mental health trust. The population covered by the trust is 1.2 million people [18]. The catchment area served by the trust is diverse, with pockets of high deprivation as well as more affluent neighbourhoods: the average index of multiple deprivation (IMD) score (28.7) is higher than England as a whole (21.67) [19]. The age demographic of the population is younger than England as a whole: 32.8% are between the ages of 18–35, compared to 24.1% in England [20]. The catchment area is highly ethnically diverse: 55% of the catchment area is represented by White ethnicity compared to 85.3% in England. The second largest ethnic group is Black/African/Caribbean/Black British, which comprises 24.7% of the local population; this is compared with only 3.5% in England. Asian ethnicity represents 10.8% of the population (including Indian, Bangladeshi, Pakistani, Chinese and other Asian), compared with 7.7% in England [21]. The unemployment rate (7.5%) is higher than that in Great Britain (6.5%) and similar to London (7.1%) [22].

### Design

Our four-year time frame allowed us to include a large amount of data as well as allowing us to capture variability associated with service change over time. The extended time frame enabled us to capture a wide breadth of individuals, experiencing acute as well as chronic mental health problems. This increases the representativeness of the sample and its generalisability to other healthcare settings.

We used the diagnostic system employed by the UK National Health Service (of which the trust is a part): the International Statistical Classification of Diseases and related health problems-10th Revision (ICD-10). We used broad ICD-10 categories (F10–19; F20–29 etc.), which allows for some subjectivity in clinical judgement whilst allowing for more specificity than a ‘psychotic/non-psychotic’ dichotomy used in other studies [8].

Linear regression was used as this increased our predictive power and did not require the use of an arbitrary cut-off (for example using the median value) for what constitutes a long stay as we are interested in those individuals with longer stays.

### Data Extract

Data was extracted from the Trust’s electronic patient record system, which ensures patient anonymity. Permission and ethical approval were obtained from the Clinical Record Interactive Search Oversight Committee, the Trust’s Corporate Audit Department and the Medical Director.

The data extract provided information on all admissions to acute adult inpatient services between 1st January 2009 to 31st December 2012 and excluded admissions to specialist services (e.g. forensic and national specialist services), child and adolescent services and older adult services. Transfers between wards were excluded from the dataset. Transfers were identified as those admissions beginning the same day or the day after the discharge date of the individual’s previous admission. This was necessary in order to avoid inadvertently counting transfers as new admissions. It’s important to note that the timeframe used to extract this data partially overlaps with that of Tulloch et al. [15] by a period of 1 year (2009).

For those predictors that change across admissions, such as diagnosis, the modal value across all admissions was used in the regression model. Where multiple model values existed the most recent value was taken.

### Analysis

Length of stay was defined as the number of days between admission date and discharge date for each admission experienced during the study period. LOS was positively skewed, with a large number of individuals with relatively short stays and a small number with very long stays. Due to the high proportions of missing data within the employment and accommodation variables (33.67%, 37.49%) a multiple imputation approach was used to create 100 imputed datasets using chained equations. These 100 datasets were then pooled to produce one complete dataset for analysis.

The data was first log-transformed so that it followed a normal distribution and then multiple linear regressions were used to explore potential predictors. Statistical effects were adjusted for the influence of other predictors in the model. The resulting coefficients represent the relationship between log-LOS and predictor variables in comparison with a reference group; positive coefficients indicate a longer LOS than the reference group; negative coefficients represent a shorter LOS than the reference group. The transformation performed well, with the exception of a number of extreme values at the low end of the distribution. The effect of the log transformation on the data is on the geometric mean, which means that exponentiated coefficients can be interpreted as multiplicative. For example an exponentiated coefficient of 2 would indicate that length of stay is expected to double for each unit increase of the predictor. The coefficients represent the change in outcome (log-LOS) for each unit increase in predictors and exponentially transformed (inverse log-transformed) coefficients represent rates e.g. a value of 1.58 meant the group had a LOS 58% longer than the reference group. The reference group was selected on the basis of it being 1) the largest or 2) most interpretable. The reference categories were as follows: female (gender); White or white British (recorded ethnicity); with partner (marital status); employed (employment status); private rental/ownership (accommodation); and psychosis (F20–29; primary diagnosis). Private rental/ownership includes patients who live independently and without any live-in support. Observations for which Cook’s distance [23] exceed 4/N, where N is the number of observations, were excluded from the analysis in order to attempt to reduce potential bias of extreme outliers that may have represented data entry errors. Heteroscedasticity was tested for with the use of a Breusch-Pagan test which was significant, prompting the use of robust standard errors.

## Results

### Sample Characteristics

A total of 7653 adults were admitted to inpatient units during the study period. There were 13,021 admissions, 5087 of which were single admissions, 2566 of which were first admissions and 5368 of which were readmissions. The mean number of readmissions per patient was 1.7 (standard deviation =1.42). The median length of stay was 19 days (interquartile range = 7–44.5), and on average patients had 3 care coordinators (mean = 2.96, standard deviation =2.49).

As can be seen in Table 1, 43.72% of the sample were female and 56.24% were male. Only 13.84% of the sample were in a relationship (including married, cohabiting and civil partnership), and only 7.34% of the sample were in employment. There was a substantial amount of missing data for employment (33.67%) and accommodation status (37.49%). For accommodation status, 42.51% lived in privately rented/owned accommodation; 10.19% of the sample lived in supported accommodation (including accommodation with criminal justice support, mental health support and sheltered housing); and 6.15% of the sample were homeless. It is important to note that the Trust’s patient record system has only pre-determined ethnicity categories, varying from broad to specific which are not compatible with the same list of ethnicities listed in UK Census data [24] (Stewart et al. 2009). In terms of recorded ethnicity, 49.7% of the sample were white or white British. The next largest group was black or black British (35.8%), followed by Asian or Asian British (6.1%), other backgrounds (4.9%), and mixed backgrounds (1.3%). The largest ICD-10 diagnostic group was psychosis (F20–29; 43.85%), followed by affective disorders (F30–39; 24.74%), personality disorders (F60–69; 8.44%) and disorders attributed to substance abuse (F10–19; 8.19%). A small proportion (3.94%) of the sample had missing data for ICD-10 diagnosis.

**Table 1:** Group sizes and % for each of the variables, including missing data categories

	Number of clients	Proportion (%)
Gender		
Female	3346	43.72
Male	4304	56.24
Missing	3	0.04
Marital status		
In a relationship	1059	13.84
Not in a relationship	6480	84.67
Missing	114	1.49
Ethnicity		
Asian or Asian British	463	6.05
Black or Black British	2735	35.76
Mixed background	100	1.31
White or White British	3798	49.66
Any other ethnic group	382	4.99
Missing	170	2.28
Employment status		
Employed	562	7.34
Other/training	228	2.98
Unemployed/retired	4286	56.00
Missing	2577	33.67
Accommodation		
Supported	780	10.19
Private rental/ ownership	3253	42.51
Council tenant	85	1.11
Homeless	471	6.15
Nursing/healthcare	181	2.37
Missing	2883	37.67
Diagnostic category (ICD-10)		
Other	20	0.26
F00–09	80	1.05
F10–19	603	7.88
F20–29	3448	45.05
F30–39	1872	24.46
F40–48	474	6.19
F50–59	30	0.39
F60–69	658	8.60
F70–79	74	0.97
F80–89	31	0.41
F90–99	30	0.39
Z	31	0.41
Missing	302	3.95

### Multiple Regression

The overall model was significant and accounted for 15% of the variance (r-squared =0.15). Table 2 shows the output from the model for the linear regression on the log transformed length of stay with outliers excluded.

**Table 2:** Output from linear regression on the log transformed length of stay excluding outliers. *p < .05; ** < .01; ***p < .001

	Coefficient (95% CI)	t-statistic
Gender (reference female)		
Male	-0.13 (−0.08 to 0.18)***	5.09
Recorded ethnicity (reference White/White British)		
Asian or Asian British	0.25 (0.15 to 0.36)***	4.86
Black or Black British	0.30 (0.24 to 0.35)***	10.78
Mixed background	0.33 (0.12 to 0.53)***	3.15
Any other background	0.08 (−0.03 to 0.19)	1.45
Marital status (reference with partner)		
Without partner	0.03 (−0.04 to 0.10)	0.88
Employment status (reference employed)		
Other/training	0.10 (−0.04 to 0.25)	1.42
Unemployed/retired	0.14 (0.05 to 0.22)***	3.25
Accommodation status (reference privately rented/owned)		
Supported accommodation	0.11 (0.03 to 0.18)***	2.86
Council tenant	-0.55 (−0.75 to −0.35)***	-5.44
Homeless	0.14 (0.04 to 0.24)**	2.68
Nursing/healthcare	0.64 (0.49 to 0.78)***	8.71
Primary diagnosis (reference F20–29)		
F00–09	0.04 (−0.19 to 0.28)	0.36
F10–19	-0.15 (−0.24 to −0.07)***	-3.45
F30–39	-0.07 (−0.13 to −0.01)*	-2.36
F40–48	-0.21 (−0.30 to −0.11)***	-4.33
F50–59	0.11 (−0.29 to 0.50)	0.53
F60–69	-0.13 (−0.21 to −0.04)***	-2.96
F70–79	-0.06 (−0.26 to 0.14)	-0.62
F80–89	-0.44 (−0.89 to 0.00)	-1.97
F90–99	0.01 (−0.39 to 0.41)	-0.05
Other	-0.08 (−0.18 to 0.02)	-1.52
Number of care coordinators	0.04 (0.02 to 0.05)***	5.62

The factors associated with lengthier stays (compared to reference categories) were recorded ethnicity (including being Asian or Asian British, Black or Black British, or having a mixed background), accommodation status (including living in supported accommodation, accommodation with nursing/healthcare, and being homeless), being male, being unemployed/retired and having a higher number of different care coordinators.

As can be seen in Table 2, Factors associated with shorter stays (compared to reference categories) were being a council tenant, having a diagnosis of a disorder due to psychoactive substance abuse (F10–19), affective disorders (F30–39), neurotic, stress-related and somatoform disorders (F40–48) and personality disorders (F60–69). Marital status was non-significant in the model.

## Discussion

### Main Findings

The main findings of this study were that a diagnosis of psychosis (F20–29), being male, being unemployed/retired, living in supported accommodation, accommodation with nursing/healthcare or being homeless, and being registered under certain ethnicity categories (particularly Caribbean) was associated with a longer LOS compared to the reference groups. Having a higher number of care coordinators was also associated with a slightly longer LOS. Being a council tenant was associated with a considerably shorter LOS compared to living in privately rented/owned housing, and marital status was not associated with LOS in this sample.

### Clinical Factors

The finding that psychosis is associated with a longer LOS replicates previous findings [8, 9, 11], with a previous paper based on a similar sample as the present study (from 2004 to 2005) finding that having a diagnosis of schizophrenia was significantly associated with longer LOS, however only severity of illness and need for rehousing remained significant in adjusted comparisons [11]. This may suggest that the effects seen in our sample may be accounted for by illness severity [9] as opposed to diagnosis alone.

The finding that having a higher number of care coordinators is associated with a slightly longer LOS might indicate that a high turnover of care coordinators is detrimental to the quality of the patient’s care, although it should be noted that the size of the effect was small. A large number of care coordinators may lead to breakdowns in communication and collaboration, leading to communication errors, poor discharge planning or inappropriate treatment, thereby prolonging a patient’s stay in hospital [25]. Alternatively, patients who stay for a longer period of time may have a higher number of care coordinators as a result of lengthy stays e.g. staff turnover either because of natural change over time, or as a result of their hard-to-manage illness. More research is needed to disentangle these possibilities.

### Demographic Factors

Our finding that being without employment is associated with a longer LOS is supported by previous studies [12, 13]. However, unemployment may simply be a ‘proxy’ for current functional impairment, as measured by scores on the Global Assessment of Functioning (GAF) [13]. The association between unemployment and longer LOS in the current study may therefore be an indication of poor occupational and social functioning, which is also reflected in need for lengthy hospitalisation. The possibility that lengthy stays in hospital in themselves lead to poor occupational and social functioning should also be considered.

It is interesting to note that a slightly earlier study (from 2007 to 2009) conducted on a similar sample found no effect of unemployment on LOS [15]. Our sample (7653) was larger than theirs (4485) which may have increased the power necessary to detect an effect in a sample where the majority is unemployed. However, the reason for this discrepancy needs to be explored further, and might again underline that idea that these predictors of LOS are likely to be temporally, as well as locality, specific.

Living in supported accommodation or living in nursing/healthcare were associated with longer LOS. Supporting previous findings by Tulloch et al. [15], we also found an association between long LOS and homelessness, though the association we found was not as strong. A number of past studies have found an effect of accommodation status on LOS [11, 14], which may have similar underlying causes such as a high level of current functional impairment.

We expected to find an effect of marital status given the finding that marital status has an effect in adequately powered studies [9], though in our sample we failed to find an effect. Since it has been suggested that marital status may be another ‘proxy’ for functional impairment, this finding may either suggest that marital status has no effect over and above other variables such as employment and accommodation status. Alternatively, it may reflect the temporal specificity of these results: the changing nature of relationships may mean that the traditional ‘single’, ‘married or cohabiting’ categories are no longer reliable indicators of relationship status or one’s current level of interpersonal functioning.

In contrast to previous studies that have found being female is associated with longer LOS, we found an association between being male and longer LOS. Tulloch et al. [9] found that the effect of female gender was apparent in samples of over 3000, which suggests that we ought to have found an effect of female gender in the model. London has been found to have a higher rate of admissions for schizophrenia and related psychoses, compared to the rest of the UK [26], and given that schizophrenia is more common in males [27, 28] this may have accounted for the association seen in this study.

Ethnicity has been found to have no effect on LOS [11, 29], though Tulloch et al. [11] found a significant effect of ethnicity that was found not to be independent of other effects. In the present study, Asian or Asian British, Black or Black British, and Mixed background ethnicity categories were associated with a longer LOS than White or White British recorded ethnicity. The fact that these effects were adjusted for other factors in the model including diagnosis, suggests that this effect is not simply due to an elevated incidence of schizophrenia in the British African population [30] and in migrant populations [27, 28].

### Limitations of the Study

There was a considerable amount of missing data for employment (34.43%) and accommodation status (39.49%), which we attempted to rectify through the use of multiple imputations (MI). Though MI has been found to be a valid method of dealing with extensive missing data, it’s important to note that that to address this in future research, additional areas and forms within the electronic patient record system will be included in the search criteria.

When considering diagnosis, we only looked at primary diagnosis and did not include secondary diagnosis primarily due to the very high rate of missing and inconsistent secondary diagnostic data in our dataset. Casey [31] estimates that 30–40% of outpatients and 40–50% of inpatients are now recognised as having a personality disorder, and as comorbid conditions are not often recognised in ordinary practice [32] we may have overlooked the contribution of personality disorder and other comorbid disorders to LOS.

As suggested by Tulloch et al. [9], illness severity could relate to LOS because it is related the clinician’s perceived need for hospitalisation, and so in future research this should be included if possible. The observed effects of unemployment, accommodation status and diagnosis of psychosis may be due in part to the patient’s level of illness severity and functional impairment. Measures of current functional impairment such as scores on the GAF would have helped to better understand the effects of these factors and their relationship to the individual’s current functioning.

### Future Research

Future research should continue to use large sample sizes to investigate the effects of demographic and clinical variables, in order that there is sufficient power to detect effects. Future research should consider the role of illness severity and level of functioning (e.g. GAF scores) in understanding the role of demographic and clinical factors on LOS. Studies should consider the role of interactions between variables e.g. between gender and diagnosis and the effect of methodology on results e.g. reference groups. Finally, studies should begin to explore the direction of these effects e.g. do a high number of care coordinators lead to lengthy stays or do lengthy stays lead to more care coordinators?; and importantly the mechanisms of these effects e.g. does a high number of care coordinators result in lengthy stays as a result of communication breakdowns? Such investigations would lead the way towards using these findings to directly inform how we care for this population.

### Conclusions

Understanding factors associated with lengthy stays in hospital is important in order to better understand psychiatric service use. Using a large sample size we were able to demonstrate the importance of certain demographic and clinical characteristics in predicting LOS, which may have been previously overlooked due to small sample sizes and lack of variance amongst inpatient characteristics [9, 17]. In addition, our findings concerning ethnicity suggest that certain groups are at a higher risk for lengthy stays, in contrast with previous research in London, which has found no effect of ethnicity. The fact that our results diverge with other studies in some cases underlines the fact that these findings are likely to be location and time specific, which underlines the importance of up-to-date research on this matter. Nevertheless, the large sample size, extended study period and ethnically diverse catchment area may increase the generalisability of our findings to other healthcare settings.

The fact that we were only able to account for 15% of the variance in the model suggests that demographic and clinical factors (at least those used here) cannot completely explain why some individuals are more likely to experience lengthy stays. LOS is likely to be multifactorially determined. Understanding how factors affect LOS is a complex task that is undoubtedly affected by a wide range of factors including setting and time period, length of study period, sample size and composition, method of analysis and definition of lengthy LOS.
